# Amide proton transfer-weighted magnetic resonance imaging for the differentiation of parotid gland tumors

**DOI:** 10.3389/fonc.2023.1223598

**Published:** 2023-08-18

**Authors:** Yihua Wang, Lijun Wang, Haitao Huang, Juntao Ma, Liangjie Lin, Lin Liu, Qingwei Song, Ailian Liu

**Affiliations:** ^1^ Department of Radiology, First Affiliated Hospital of Dalian Medical University, Dalian, China; ^2^ Department of Stomatology, First Affiliated Hospital of Dalian Medical University, Dalian, China; ^3^ Clinical and Technical Support, Philips Healthcare, Beijing, China

**Keywords:** parotid gland tumor, magnetic resonance imaging, amide proton transfer-weighted image, pleomorphic adenoma, Warthin tumor

## Abstract

**Purpose:**

To assess the usefulness of amide proton transfer-weighted (APTw) imaging in the differentiation of parotid gland tumors.

**Materials and methods:**

Patients with parotid gland tumors who underwent APTw imaging were retrospectively enrolled and divided into groups according to pathology. Two radiologists evaluated the APTw image quality independently, and APTw images with quality score ≥3 were enrolled. The maximum and average values of APTw imaging for tumor lesions (APTmax and APTmean) were measured. The differences in APTmax and APTmean were compared between malignant tumors (MTs) and benign tumors (BTs), as well as between MTs and pleomorphic adenomas (PAs) and between MTs and Warthin tumors (WTs). Independent-samples *t*-test, Kruskal–Wallis *H* test, and receiver operating characteristic (ROC) curve analyses were used for statistical analysis.

**Results:**

Seventy-three patients were included for image quality evaluation. In this study, 32/73 and 29/73 parotid tumors were scored as 4 and 3, respectively. After excluding lesions with quality score ≤2 (12/73), the APTmean and APTmax of MTs were 4.15% ± 1.33% and 7.43% ± 1.61%, higher than those of BTs 2.74% ± 1.04% and 5.25% ± 1.54%, respectively (*p* < 0.05). The areas under the ROC curve (AUCs) of the APTmean and APTmax for differentiation between MTs and BTs were 0.819 and 0.821, respectively. MTs indicated significantly higher APTmean and APTmax values than those of PAs (*p* < 0.05) and WTs (*p* < 0.05). The AUCs of the APTmean and APTmax for differentiation between MTs and PAs were 0.830 and 0.815 and between MTs and WTs were 0.847 and 0.920, respectively.

**Conclusion:**

Most APTw images for parotid tumors had acceptable image quality for APTw value evaluation. Both APTmax and APTmean can be used to differentiate MTs from BTs and to differentiate MTs from subtype parotid gland tumors.

## Introduction

Salivary gland tumors account for approximately 2%~5% of all tumors in the head and neck (1, 2), with nearly 80% occurring in the parotid glands. The parotid benign tumors (BTs) and malignant tumors (MTs) account for approximately 80% and 20%, respectively (3). For BTs, local parotidectomy or superficial lobectomy is adopted to protect the facial nerve. For MTs, total parotidectomy is required (4, 5). Preoperative biopsy is helpful for the qualitative diagnosis of parotid gland tumors, but some punctures have the risk of capsule rupture, which will greatly increase the risk of tumor proliferation or implantation (6). Therefore, noninvasive qualitative preoperative diagnosis has become an urgent clinical need.

Magnetic resonance imaging (MRI) is one of the major methods to diagnose tumors of the head and neck with good visualization (7, 8). However, the pathological types of parotid gland tumors are various, and there exists substantial overlap in the appearance of tumors, which limits the role of conventional MRI in characterization and brings great difficulty to the preoperative qualitative diagnosis (9). In the past, diffusion-weighted imaging (DWI), dynamic contrast-enhanced MRI (DCE-MRI), and other functional imaging have been used to evaluate parotid tumors, but the diagnostic ability of one single functional MRI technology is limited (10–12). Rather, multiparametric analysis is usually required to improve diagnostic accuracy (13, 14). However, there are still some challenges in the clinical applications of multiparametric analysis because of the long acquisition time and requirement in the injection of contrast agents in DCE-MRI.

Amide proton transfer-weighted (APTw) imaging is a novel imaging technique that uses endogenous contrast by chemical exchange saturation transfer to indirectly detect mobile proteins and peptides in tissues, which are thought to closely relate to tumor metabolism (15). The clinical utility of APTw imaging has already been demonstrated in glioma, lung cancer, prostate cancer, endometrial carcinoma, and rectal cancer (16–19). Kamitani et al. (20) demonstrated that for parotid tumors, the mean APTw values measured from circle regions of interest (ROIs) in MTs were higher than those in BTs. Bae et al. (21) reported about parotid gland that APTw imaging was superior to conventional MRI contrasts and to advanced functional imaging methods such as DCE-MRI and DWI. However, one limitation of APTw is its vulnerability to hyperintensity artifacts in the parotid gland, resulting in false positives in the evaluation of lesions probably (22, 23). Therefore, in this study, we investigated APTw imaging in parotid lesions in terms of image quality to ensure the accuracy of APTw measurements and evaluated its ability to differentiate among parotid gland tumors.

## Materials and methods

### Patients

**Figure 1:**
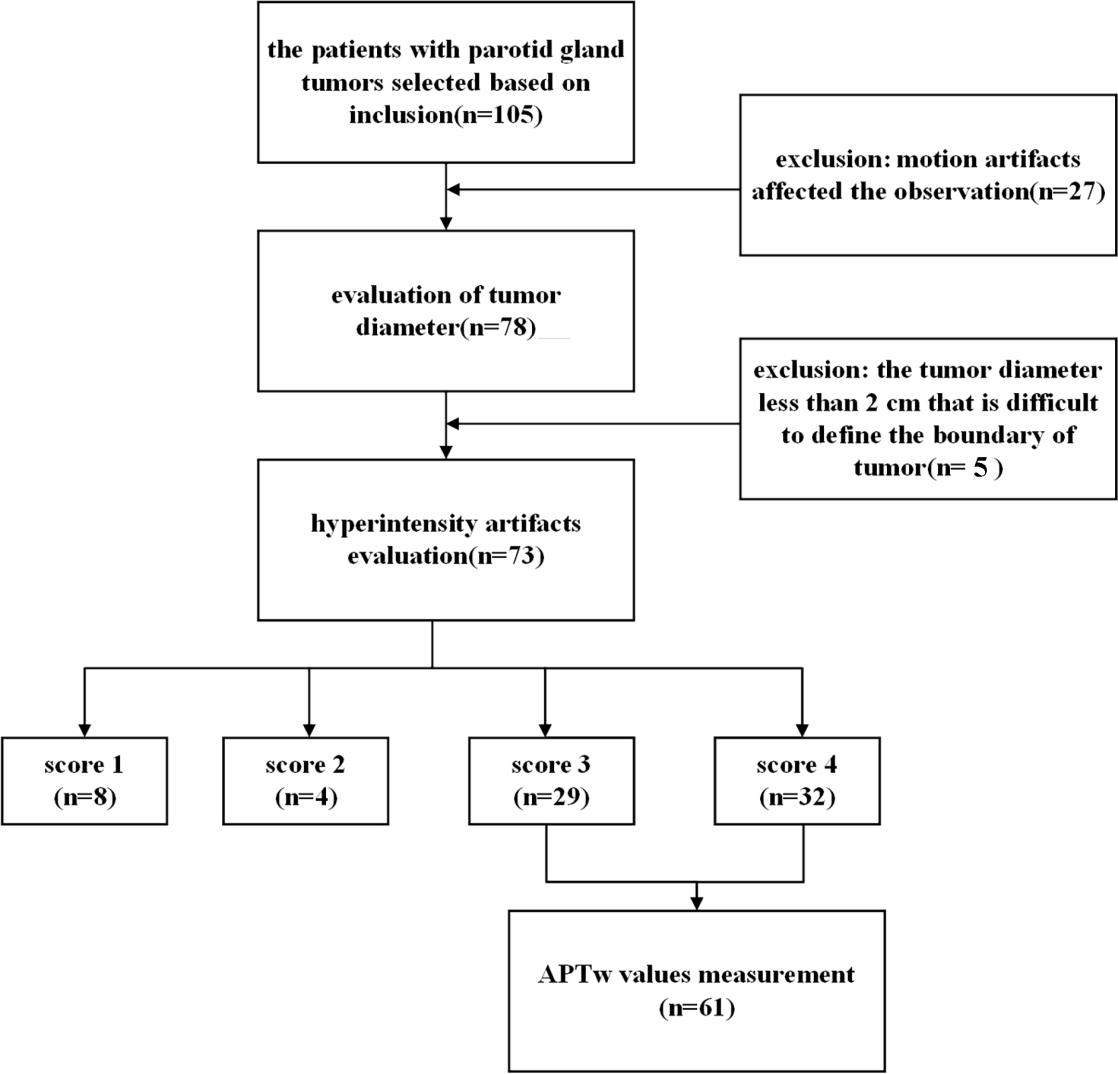
Flowchart depicting the patient selection.

The institutional review board of our hospital approved our retrospective study (license number: PJ-KS-XJS-2021-18). The patients who participated in this study provided their written informed consent. Inclusion criteria were as follows: 1) the clinical and pathological information was complete; 2) 3.0T MRI examination [including T1-weighted imaging (T1WI), T2-weighted imaging (T2WI), and APTw imaging] was performed within 1 week before treatment; 3) no treatment before MRI examination. Exclusion criteria were as follows: 1) parotid lesions were not clearly visible on images or motion artifacts affected the observation; 2) the tumor diameter was less than 2 cm that it was difficult to define the boundary of the tumor. The flowchart of patient inclusion and exclusion is shown in **Figure 1**.

Based on inclusion criteria, the imaging and clinical information data of 105 patients with parotid gland tumors in our hospital from September 2020 to October 2022 were retrospectively analyzed. All patients underwent surgical treatment in the Department of Stomatology of our hospital within 1 week after MRI examination. All extracted tumor tissues routinely underwent histopathological examination after the operation. These tissues were embedded in paraffin, stained with hematoxylin–eosin, and examined microscopically.

### MRI

APTw imaging was performed using a 3.0T MR scanner (Ingenia CX; Philips Healthcare, Best, Netherlands) with a 32-channel phase-array head coil. In addition, the protocol of conventional MRI was acquired, including the axial T1WI and axial fat-suppressed T2WI. The APTw sequence used in this study is based on Chen et al. (23). The detailed parameters of all MRI sequences are shown in **Table 1**. The MTR_asym(3.5 ppm)_ was calculated by the following equation: MTR_asym(3.5 ppm)_ = (S_-3.5 ppm_ − S_3.5 ppm_)/S_0_, where S_-3.5 ppm_ is the signal intensity acquired at the saturation frequency of -3.5 ppm, S_3.5 ppm_ is the signal intensity acquired at the saturation frequency of 3.5 ppm, and S_0_ is the reference signal intensity acquired at a saturation frequency of 1,540 ppm (the water frequency was referred to as 0 ppm).

**Table 1:** Scan parameters of T1WI, T2WI, and APTw.

	TR (ms)	TE (ms)	Voxel (mm)	FOV (mm)	Matrix
**T_1_WI**	466	8.1	0.55×0.72×4	200×200×89	364×257×18
**T_2_WI**	2,122	112	0.7×0.7×4	300×300×89	428×428×18
**APTw**	3,000	7.9	2.5×2.5×2.5	230×221×62	120×140×40

APTw, amide proton transfer-weighted imaging; T1WI, T1-weighted imaging; T2WI,T2-weighted imaging; TR, repetition time; TE, echo time.

### MR image evaluation

APTw images were automatically reconstructed after data acquisition and then transferred to the Intellispace Portal (ISP v9.0, Philips Healthcare) workstation. The image quality evaluation and quantitative measurements of APTw image were implemented by two experienced radiologists in MRI diagnosis independently (radiologist 1 and radiologist 2 had 3 and 20 years of MRI diagnosis experience, respectively) who were blinded to pathological results. With APTw images fused to axial T2WI images, the degree of image quality was judged with a 4-scale scoring system according to a previous report (23): 4 = excellent, tumor could be recognized on APTw images without hyperintensity artifacts; 3 = good, hyperintensity artifacts impair less than 50% tumor; 2 = moderate, hyperintensity artifacts impair more than 50% tumor; 1 = poor, the entire tumor is impaired by hyperintensity artifacts.

**Figure 2:**
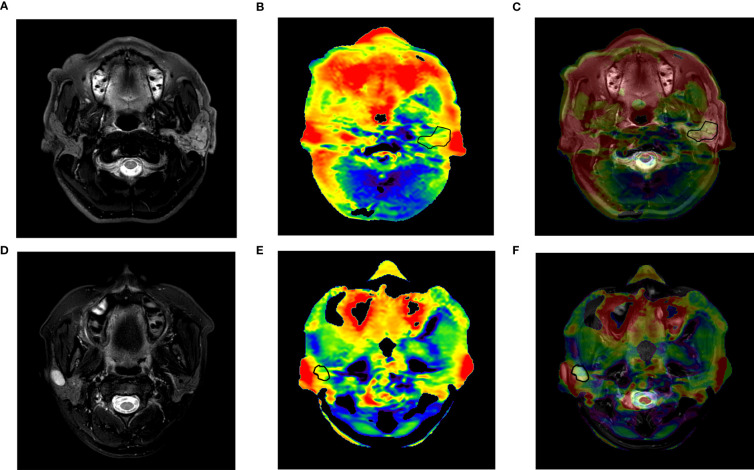
**(A–C)** A 57-year-old man with adenoid cystic carcinoma of the left parotid gland. **(A)** T2WI; **(B)** APTw image; **(C)** APTw image (fused on T2WI) showed an image quality score of 3 with little hyperintensity artifact less than 50% tumor, an APTmean of 5.14%, and an APTmax of 6.75%. **(D–F)** A 30-year-old woman with pleomorphic adenoma of the right parotid gland. **(D)** T2WI; **(E)** APTw image; **(F)** APTw image (fused on T2WI) showed an image quality score of 4, an APTmean value of 1.95%, and an APTmax of 4.9%. APTmax, the maximum value of APTw imaging; APTmean, the average value of APTw imaging; APTw, amide proton transfer-weighted.

APTw images with image quality score no higher than 2 were excluded for further analyses. The ROI was carefully drawn on a slice of the fused image showing the maximum lesion to cover the solid part of the tumor as much as possible and exclude the cystic degeneration, necrosis, and hyperintensity artifacts from surrounding tissues (**Figure 2**). The maximum (APTmax) and the average (APTmean) values were recorded.

### Statistical analysis

For statistical analyses of patient information and diagnostic efficacy of APTw, we used SPSS (version 25.0; IBM Corp., Armonk, NY, USA) and MedCalc (version 20 MedCalc Software Ltd., Ostend, Belgium). The interobserver reliability for all APTw values measured by two radiologists was assessed *via* intraclass correlation coefficient (ICC) (excellent, >0.75; good, 0.60~0.74; fair, 0.40~0.59; poor, <0.40). One-sample Kolmogorov–Smirnov test was performed to test the normality of APTmax and APTmean values for both BTs and MTs, as well as patient ages. When continuous variables conformed to the normal distribution, the parameters were expressed as mean ± standard deviation, and independent-samples *t*-test was used for comparisons between BTs and MTs groups; otherwise, they were expressed as median (first quartile, third quartile), and Mann–Whitney U test was used. The Kruskal–Wallis *H* test was used to test the differences of the two parameters among pleomorphic adenomas (PAs), Warthin tumors (WTs), and MTs. The pairwise comparison with Bonferroni correction was made with overall test statistically significant for the above three groups. Receiver operating characteristic (ROC) curve analysis was used to determine the diagnostic value of APTmax and APTmean for the differentiation between MTs and BTs. The threshold criterion was calculated to maximize the Youden index. ROC curves were compared by the method of DeLong et al. (24). *p* < 0.05 was considered statistically significant.

## Results

### Patient characteristics

Among the included 105 patients, 27 patients were excluded because of the incomplete MR scans or severe motion artifacts on images, and five patients were excluded due to the small tumor size and unclear tumor boundary. Finally, we enrolled a total of 73 patients for the next image quality analysis. According to the benign and malignant pathological results, 73 patients who were included for image quality scale analysis were divided into two groups (**Table 2**). There was no significant difference in age (*p* = 0.63) between benign and malignant groups.

**Table 2:** Demographics for patients confirmed by surgery.

	Benign group (n=62)	Malignant group (n=11)
**Male : Female**	41:21	8:3
**Age (years)**	25-85 (mean 55.73 ± 15.38)	46-82 (mean 58.00 ± 10.95)
**Pathology**	32 pleomorphic adenoma	3 mucoepidermoid carcinoma
	21 Warthin tumor	2 acinic cell carcinoma
	8 base cell adenoma	2 adnoid cystic carcinoma
	1 schwannoma	1 salivary duct carcinoma
		1 malignant neurofibroma
		1 non Hodgkin's lymphoma
		1 poorly differentiated carcinoma

### Score of APTw image quality

Interobserver agreement was excellent, with ICCs = 0.989 for artifact scores of parotid lesions by the two readers. In the evaluation of image quality, 32 out of 73 parotid tumors (43.84%) were considered for score 4, and 29 out of 73 (39.73%) for score 3. Moreover, 5.48% (4/73) and 10.95% (8/73) of tumors were scored 2 and 1, respectively, which showed parotid lesions highly affected by hyperintensity artifacts and were removed in the subsequent measurement of APTw values.

### APTw finding and diagnostic performance between BTs and MTs

**Figure 3:**
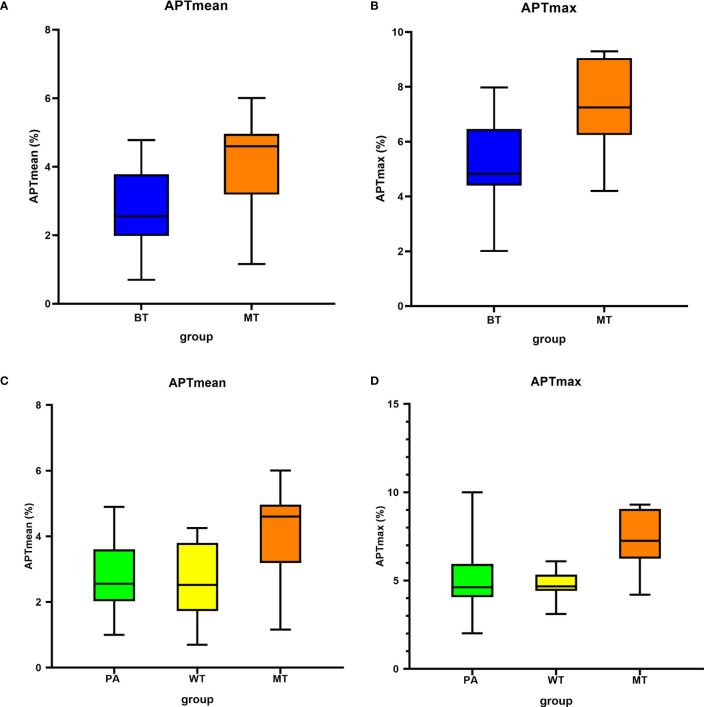
Box plots show the comparison of APTmean and APTmax among groups. Line in box represents the median, and the height of the box represents the interquartile range. **(A, B)** Comparison between BT and MT; **(C, D)** comparison among PA, WT, and MT. APTmax, the maximum value of APTw imaging; APTmean, the average value of APTw imaging; BT, benign tumor; MT, malignant tumor; PA, pleomorphic adenoma; WT, Warthin tumor.

After excluding the cases with image quality scores ≤2, 61 patients were involved in the quantitative evaluation. Interobserver agreement was excellent, with ICCs = 0.994 and 0.918, respectively, for APTmax and APTmean measurements, and the average values by the two observers were taken for analyses. The APTmean of MTs (4.15% ± 1.33%) was significantly higher than that of BTs (2.74% ± 1.04%) (*p* < 0.05), and the APTmax value of MTs was (7.43% ± 1.61%), similarly higher than that of BTs (5.25% ± 1.54%) (*p* < 0.05) (**Table 3**, **Figure 3**).

**Table 3:** Comparison of APTw values between BT and MT.

	ICC^*^	MT(n=11)	BT(n=50)	*p*
**APTmax(%)**	0.918	7.43 ± 1.61	5.25 ± 1.54	<0.01
**APTmean(%)**	0.994	4.15 ± 1.33	2.74 ± 1.04	<0.01

*Interobserver agreement was excellent.

APTmax, the maximum value of APTw imaging; APTmean, the average value of APTw imaging; APTw, amide proton transfer-weighted; BT, benign tumor; MT, malignant tumor.

**Figure 4:**
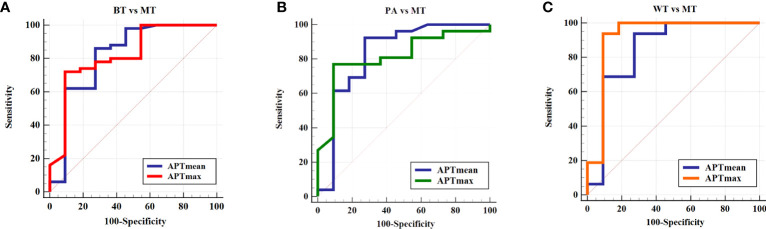
ROC curve of the APTmean and APTmax for differentiation between **(A)** BT and MT, **(B)** PA and MT, and **(C)** WT and MT. APTmax, the maximum value of APTw imaging; APTmean, the average value of APTw imaging; BT, benign tumor; MT, malignant tumor; PA, pleomorphic adenoma; WT, Warthin tumor.

The threshold of APTmean was 3.98%, and its area under the ROC curve (AUC), sensitivity, and specificity were 0.819, 86.00%, and 72.73%, respectively, for differential diagnosis between BTs and MTs. Moreover, ROC curve analysis indicated that an APTmax of 5.9% was the optimum threshold to distinguish between BTs and MTs, with AUC, sensitivity, and specificity of 0.821, 53.33%, and 82.61%, respectively. There was no significant difference in diagnostic efficacy between the above parameters (Z = 0.017, *p* = 0.987) (**Table 4**, **Figure 4**).

**Table 4:** The ROC curve of the values of APTw to differentiate between groups.

	Cut off value	AUC (95% confidence interval)	Sen(%)	Sp(%)	PPV(%)	NPV(%)
BT vs. MT						
**APTmax**	5.9%	0.821 (0.702, 0.907)	53.33	82.61	41.67	97.30
**APTmean**	3.98%	0.819 (0.700, 0.906)	86.00	72.73	53.34	93.48
PA vs. MT						
**APTmax**	5.65%	0.815 (0.653, 0.923)	76.90	90.90	62.50	95.24
**APTmean**	3.98%	0.830 (0.671, 0.933)	92.31	72.73	80.00	88.89
WT vs. MT						
**APTmax**	5.50%	0.920 (0.750, 0.989)	93.75	90.91	90.91	93.75
**APTmean**	3.90%	0.847 (0.656, 0.955)	93.57	72.73	88.89	83.33

AUC, area under the receiver operating characteristic curve; APTmax, the maximum value of APTw imaging; APTmean, the average value of APTw imaging; APTw, amide proton transfer-weighted; BT, benign tumor; MT, malignant tumor; NPV, negative predictive value; PA, pleomorphic adenoma; PPV, positive predictive value; Sen, sensitivity; Sp, specificity; WT, Warthin tumor.

### APTw finding and diagnostic performance among PAs, WTs, and MTs

There were significant differences among these three groups (APTmean, *p* = 0.03; APTmax, *p* = 0.02). The pairwise comparisons showed that the APTmean and APTmax values in MTs were significantly higher than those of PAs and WTs, while the difference of the two parameters between PAs and WTs was not significant (*p* > 0.99, **Table 5**).

**Table 5:** Comparison of APTw values among PA, WT, and MT.

	PA(n=26)	WT(n=15)	MT(n=11)	*p*	*p*		
					PA vs. WT	PA vs. MT	WT vs. MT
**APTmax(%)**	5.11 ± 1.88	4.67 ± 0.83	7.43 ± 1.61	0.02*	>0.99	<0.01*	<0.01*
**APTmean(%)**	2.69 ± 0.95	2.58 ± 1.06	4.15 ± 1.33	0.03*	>0.99	<0.01*	<0.01*

*p < 0.05.

APTmax, the maximum value of APTw imaging; APTmean, the average value of APTw imaging; APTw, amide proton transfer-weighted; BT, benign tumor; MT, malignant tumor; PA, pleomorphic adenoma; WT, Warthin tumor.

The diagnostic performance of APTmean and APTmax for differentiating among these three groups was shown in **Tables 4** and **5**. When the thresholds of the APTmean and APTmax were 3.98% and 5.65%, respectively, the optimal diagnostic performance for differentiating between PAs and MTs can be achieved. The AUC, sensitivity, and specificity of the APTmean between PAs and MTs were 0.830, 92.31%, and 72.73%. And the AUC, sensitivity, and specificity of the APTmax between PAs and MTs were 0.815, 76.90%, and 90.90%. Meanwhile, the threshold APTmean value of 3.90% can be used for optimal differential diagnosis between WTs and MTs with AUC, sensitivity, and specificity 0.847, 93.57%, and 72.73%. And the threshold APTmax value of 5.50% can be used for differentiating between WTs and MTs with AUC, sensitivity, and specificity 0.920, 93.75%, and 90.91%. There was no significant difference in diagnostic efficacy between APTmean and APTmax (PAs and MTs: Z = 0.141, *p* = 0.887; WTs and MTs: Z = 0.707, *p* = 0.479).

## Discussion

In this study, we investigated the image quality of APTw imaging of parotid gland tumors and evaluated the characteristics and diagnostic performance of APTmax and APTmean. APTmean and APTmax in MTs were higher than those in BTs with high diagnostic efficacy. However, APTw imaging based on the current technology may be associated with severe artifacts in parotid glands (16.43% of cases), which can affect the evaluation of tumors.

In the parotid gland, APTw hyperintensity artifacts, diffused from the bone, air, ear, and other surrounding tissues, can affect the display of peripheral lesions and thus the quantitative APTw measurements. In this study, most of the cases with score ≤2 were PAs and WTs, where more than half of the tumors and the surrounding normal parotid gland parenchyma showed significantly hyperintensity. These hyperintensity artifacts were usually spread from the ear and mandible regions around the parotid gland. On the other hand, the area of hemorrhage, necrosis, and cystic degeneration can also contribute to the increase of APTw values due to the increase of mobile water molecule and amide protons. Chen et al. (23) demonstrated that approximately 70.6% of parotid gland lesions had no or small artifacts and the APTw measurements of the lesion would be reliable after excluding cases with poor image integrity and severe artifacts. Takeshi et al. (20) evaluated the difference in APTw values of BTs and MTs by sketching three circular ROIs in the parenchyma of parotid tumors. This measurement method can avoid artifacts of necrosis and cystic degeneration but cannot determine the maximum value of the whole tumors. Therefore, this study evaluated the image quality (83.57% of cases with an acceptable image quality score of 3 or 4) before the overall measurement of lesions, excluded lesions with severe artifacts, and determined reliable cases for analyses. The fusion of APTw with conventional structural images can be helpful for the determination of tumor boundary.

APTw imaging has been widely used in the assessment of tumor metabolism, ischemic penumbra of cerebral infarction, neurodegenerative changes, etc. (25, 26). In previous studies (27), it was found that MTs showed generally higher APTw values due to increased mobile proteins and polypeptides; however, abundant new blood vessels and increased vascular permeability could also lead to significantly increased APTw signal intensity in BTs. Most studies (20, 28) used the mean APTw value of the lesion to evaluate the overall lesion. Generally, the APTw mean value of MTs is higher than that of BTs, and similar results were also observed in this study. However, in the study of Ochiai et al. (29) on the evaluation of endometrial carcinoma, APTmean values had no significant difference between type I and type II endometrial carcinoma, but APTmax was significantly higher in type II carcinomas than that in type I, which may be due to the heterogeneity of lesion histology. APTmax might indicate the position with the most active metabolism and the highest cell density, which can evaluate heterogeneity of tumors more accurately in some studies (30, 31). The malignant lesions with more active cell proliferation, which showed a higher APTw value, may be related to the capacity of tumor invasion and prognosis. In the study by Law et al. (27), the AUC and sensitivity of the APTw value at the 90th percentage in head and neck tumors were significantly higher than those of the mean APTw value. Therefore, we speculated that the maximum APTw value of the whole lesions may have high diagnostic efficacy between BTs and MTs.

In this study, we excluded the cases showing artifacts that might affect the APTw value measurements and selected the slice with the largest section of lesion for ROI delineation to obtain the mean and maximum APTw values. It was found that in this study, the AUC of the APTmax value was similar to that of the APTmean for differentiation between MTs and BTs, but the APTmax was more specific than the APTmean. Additionally, because of hypercellularity, some functional MRI sequences showed image overlap between WTs and MTs (32, 33). In this study, the APTmean also showed false-positive results in the evaluation of MTs, but the APTmax values in the two groups of tumors have less overlap with higher diagnostic efficiency, which may suggest that the APTmax can play a complementary role in the differential diagnosis of parotid gland tumors. Although the overall APTmean and APTmax of BTs were lower, not a few cases of PA exhibited high APTmax signals. Some studies (20, 34, 35) believe that the epithelial and interstitial components of PA are diverse, and mesenchymal-like component is rich in mucoid, which can cause the variety of APTw values and might be the interference for APTw in parotid gland tumor evaluation. Moreover, some MTs (36) were low-grade malignant (such as mucoepidermoid carcinomas), and the increase of cell proliferation was not obvious, with the increase of the APTw value inapparent, which can be difficult to be distinguished from some BTs with more active proliferation. Therefore, the pathological complexity of parotid gland tumors mentioned above led to their diverse imaging manifestations, which also affected the accuracy of the APTmean and APTmax in the diagnosis of parotid gland tumors.

There are some limitations in our study. First, we did not choose the overall volume but the largest slice of tumor for analysis, which may affect the evaluation of tumor heterogeneity. Second, our study did not register imaging results with pathological findings accurately. It is necessary to further explore the relationship between the heterogeneity of imaging manifestations and tissue structure. Third, some previous studies (11, 13) demonstrated a high diagnostic performance with the combination of DCE-MRI and DWI, and this study did not compare APTw-MRI with other functional imaging sequences and investigate whether APTw can further improve the diagnostic performance of other functional sequences. Fourth, this was a small-sample retrospective study, especially, with few other BT types except PAs and WTs, and the malignant group was a small sample and includes different histological patterns. The pathological types were relatively limited, which may cause some errors in the statistics of the results. The low disease prevalence of MTs is the fundamental limiting factor. It is recommended to conduct further research to confirm the clinical impact of our results and the differences between specific sites in a larger cohort.

## Conclusion

Both APTmax and APTmean values can differentiate benign and malignant parotid gland tumors, which suggested that APTw imaging might be helpful in the differentiation of benign and malignant parotid tumors before surgery. In our study, most APTw images in parotid gland tumors (83.57%) had acceptable image quality for APTw value evaluation. However, the technique still needs to be improved for reduced image artifacts.

## Data availability statement

The raw data supporting the conclusions of this article will be made available by the authors without undue reservation.

## Ethics statement

The studies involving human participants were reviewed and approved by the Ethics Committee of the First Affiliated Hospital of Dalian Medical University (license number: PJ−KS−XJS−2021-18). The patients/participants provided their written informed consent to participate in this study.

## Author contributions

YW: methodology, investigation, data curation, writing—original draft, and writing—review and editing. LW: conceptualization, investigation, writing—review and editing, supervision, and project administration. LJL: software, visualization, and writing—review and editing. HH, JM, and LL: investigation, resources, and data curation. QS: resources and data curation. AL: supervision and project administration. All authors contributed to the article and approved the submitted version.
